# Long-term outcomes following vehicle trauma related acute kidney injury requiring renal replacement therapy: a nationwide population study

**DOI:** 10.1038/s41598-020-77556-3

**Published:** 2020-11-25

**Authors:** Chieh-Kai Chan, Chun-Yi Chi, Tai-Shuan Lai, Tao-Min Huang, Nai-Kuan Chou, Yi-Ping Huang, John R. Prowle, Vin-Cent Wu, Yung-Ming Chen

**Affiliations:** 1 Department of Internal Medicine, National Taiwan University Hospital, Hsin-Chu Branch, Hsin Chu County, Taiwan; 2 Department of Internal Medicine, National Taiwan University Hospital, 7 Chung-Shan South Road, Zhong-Zheng District, Taipei, 100 Taiwan; 3 Graduate Institute of Clinical Medicine, College of Medicine, National Taiwan University, Taipei, Taiwan; 4 Department of Internal Medicine, National Taiwan University Hospital, Yun-Lin Branch, Yun Lin County, Taiwan; 5 Department of Surgery, National Taiwan University Hospital, Taipei, Taiwan; 6 National Taiwan University Hospital Study Group on Acute Renal Failure, Taipei, Taiwan; 7 Adult Critical Care Unit, The Royal London Hospital, Barts Health NHS Trust, London, UK; 8 Department of Renal Medicine and Transplantation, The Royal London Hospital, Barts Health NHS Trust, London, UK; 9 William Harvey Research Institute, Queen Mary University of London, London, UK

**Keywords:** Haemodialysis, Outcomes research

## Abstract

Acute kidney injury (AKI) is a frequent complication of traumatic injury; however, long-term outcomes such as mortality and end-stage kidney disease (ESKD) have been rarely reported in this important patient population. We compared the long-term outcome of vehicle-traumatic and non-traumatic AKI requiring renal replacement therapy (AKI-RRT). This nationwide cohort study used data from the Taiwan National Health Insurance Research Database. Vehicle-trauma patients who were suffered from vehicle accidents developing AKI-RRT during hospitalization were identified, and matching non-traumatic AKI-RRT patients were identified between 2000 and 2010. The incidences of ESKD, 30-day, and long-term mortality were evaluated, and clinical and demographic associations with these outcomes were identified using Cox proportional hazards regression models. 546 vehicle-traumatic AKI-RRT patients, median age 47.6 years (interquartile range: 29.0–64.3) and 76.4% male, were identified. Compared to non-traumatic AKI-RRT, vehicle-traumatic AKI-RRT patients had longer length of stay in hospital [median (IQR):15 (5–34) days vs. 6 (3–11) days; *p* < 0.001). After propensity matching with non-traumatic AKI-RRT cases with similar demographic and clinical characteristics. Vehicle-traumatic AKI-RRT patients had lower rates of long-term mortality (adjusted hazard ratio (HR), 0.473; 95% CI, 0.392–0.571; *p* < 0.001), but similar rates of ESKD (HR, 1.166; 95% CI, 0.829–1.638; *p* = 0.377) and short-term risk of death (HR, 1.134; 95% CI, 0.894–1.438; *p* = 0.301) as non-traumatic AKI-RRT patients. In competing risk models that focused on ESKD, vehicle-traumatic AKI-RRT patients were associated with lower ESKD rates (HR, 0.552; 95% CI, 0.325–0.937; *p* = 0.028) than non-traumatic AKI-RRT patients. Despite severe injuries, vehicle-traumatic AKI-RRT patients had better long-term survival than non-traumatic AKI-RRT patients, but a similar risk of ESKD. Our results provide a better understanding of long-term outcomes after vehicle-traumatic AKI-RRT.

## Introduction

### Background

Acute kidney injury (AKI) has been studied in a wide range of populations and has been consistently associated with increased risk of future morbidity and mortality^1,2^. The in-hospital mortality associated with AKI ranges from 9.1% to 21.9% in the United States^3^, and amounts to as high as 62% in the critical care setting in patients requiring renal replacement therapy (RRT)^4,5^. As AKI is a clinical syndrome it treatment should be tailored to its underlying etiology. While considerable data exist on the epidemiology, outcomes and suggested management of AKI in settings such as sepsis or nephrotoxin exposure, comparatively less data is available to guide clinicians on the clinical course of AKI after major trauma.

Trauma is a leading cause of hospitalization worldwide, which mainly affects a young and previously healthy population both in the developed and developing world. The incidence of AKI among major traumatic injuries has been reported to be 6.0–36.8%^6,7^, and traumatic AKI has been associated with a mortality rate of 14.9–57.0%^7–10^. Overall around 5% of trauma patients admitted to the intensive care unit (ICU) require RRT^10^. In a recent systematic review the pooled incidence of AKI after trauma was 20.4% with an associated 3.6-fold increase in the relative risk of death, commonly reported associations of AKI after trauma include older age, higher severity of injury, volume of blood transfusion, abdominal site of injury and presence of comorbid disease including diabetes mellitus^11^. Prior studies of traumatic AKI-RRT have focused mainly on short-term outcomes^12,13^ and detailed comparisons of outcomes between traumatic-AKI and AKI of differing etiologies that have not been made.

Given the paucity of studies concerning traumatic AKI-RRT, we sought to describe its epidemiology and associated short and long-term outcomes in a large epidemiological database.

## Results

### Temporal change in in-hospital mortality

**Figure 1:**
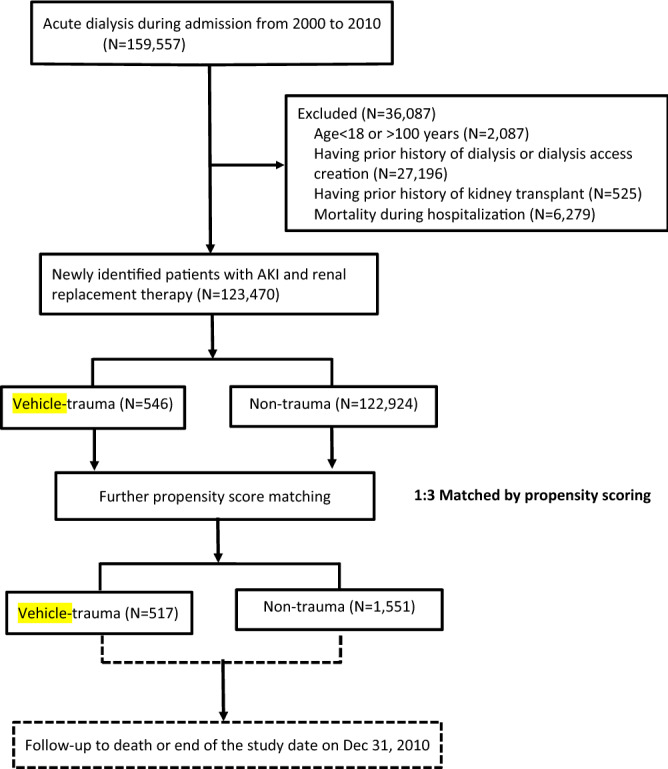
Flow chart of the participants.

**Figure 2:**
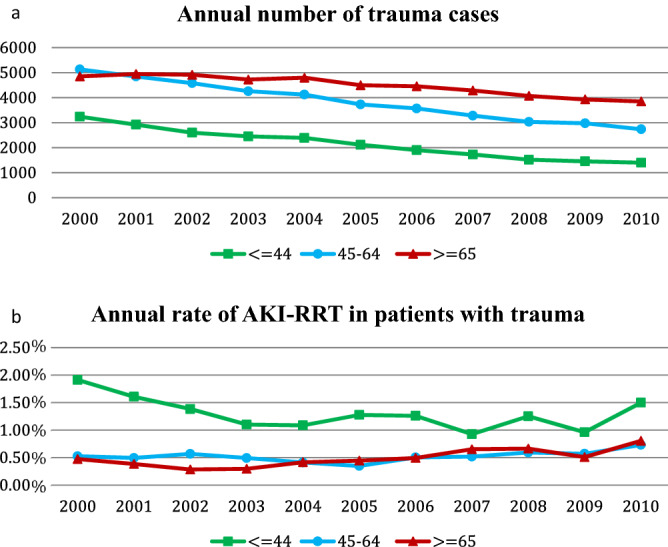
Temporal patterns of annual number of trauma cases (**a**) and annual rate of vehicle-traumatic AKI-RRT (**b**) stratified by age groups (annual rate of vehicle-traumatic AKI-RRT = annual number of vehicle-traumatic AKI-RRT/ annual number of trauma cases). (**b**) *p* for trend, age ≦ 44: 0.073, 45 < age < 65: 0.183, age ≧ 65: 0.004).

**Figure 3:**
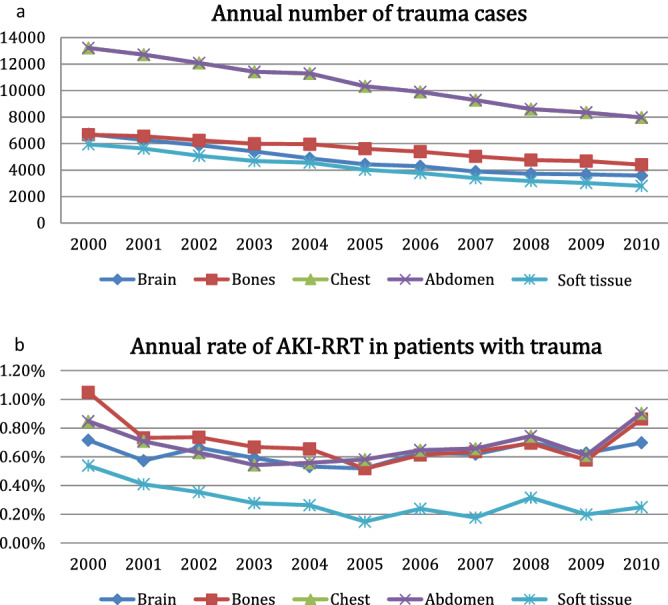
Temporal patterns of annual number of trauma cases (**a**) and annual rate of traumatic AKI-RRT (**b**) stratified by injured body part (annual rate of vehicle-traumatic AKI-RRT = annual number of vehicle-traumatic AKI-RRT/ annual number of trauma cases). (**b**) *p* for trend, brain: 0.697, bones: 0.186, chest: 0.484, abdomen: 0.484, Skin: 0.024).

Among 123,470 hospitalized AKI-RRT patients in the past decade, we identified 546 vehicle-traumatic AKI-RRT patients who survived to index discharge (Fig. 1). The annual number of trauma cases decreased gradually; however, the proportions of vehicle-traumatic AKI-RRT patients tended to increase over time, especially in the elderly group (aged ≥ 65 years) (Fig. 2). In our cohort, young patients (aged ≤ 44 years) were less likely to be admitted because of trauma; however, they had the highest incidence of vehicle-traumatic AKI-RRT (Fig. 2) during hospitalization. On the other hand, soft tissue injury from trauma had the lowest incidence of AKI-RRT (Fig. 3).

### Patient characteristics before and after propensity score matching

Compared to patients with other causes of AKI-RRT, vehicle-traumatic AKI-RRT patients were younger [median IQR: 47.6 (29.0–64.3) vs. 65.9 (53.7–74.5), *p* < 0.001) and more of them were male (77.1% vs. 54.2%, *p* < 0.001). Vehicle-traumatic AKI-RRT patients also had a lower rate of cerebrovascular disease, COPD, diabetes, liver disease, and advanced chronic kidney disease (CKD) which was defined as the presence of CKD coding and concomitant reimbursement coding of erythropoiesis-stimulating agents without copayment. More pulmonary and cardiac complications occurred in AKI-RRT patients than non-traumatic AKI-RRT patients during hospitalization. Among them, 517 vehicle-traumatic AKI-RRT patients were successfully matched with 1,551 non-traumatic AKI-RRT patients (Table 1).

**Table 1:** Clinical characteristics after propensity score matching (NHIR database).

Variables	Before matching			After matching		
	Non-trauma group (n = 122,924)	Trauma group (n = 546)	*p* Value	Non-trauma group (n = 1,551)	Trauma group (n = 517)	*p* Value
Gender (Male)	66,595 (54.2%)	421 (77.1%)	< 0.001	1183 (76.3%)	395 (76.4%)	0.952
Age (year) (IQR)	65.9 (53.7–74.5)	47.6 (29.0–64.3)	< 0.001	48.7 (34.2–64.9)	48.7 (31.5–65.3)	0.469
**Pre-admission Comorbidities**						
Charlson comorbidity index (IQR)	0 (0–0)	0 (0–1)	< 0.001	0 (0–1)	0 (0–1)	0.075
Myocardial infarction	988 (0.8%)	3 (0.6%)	0.51	10 (0.6%)	3 (0.6%)	0.872
Congestive heart failure	5410 (4.4%)	22 (4.0%)	0.67	80 (5.1%)	22 (4.3%)	0.412
Cerebrovascular disease	9742 (7.9%)	13 (2.4%)	< 0.001	41 (2.6%)	13 (2.5%)	0.874
Dementia	1384 (1.1%)	1 (0.2%)	0.04	6 (0.4%)	1 (0.2%)	0.512
Chronic obstructive pulmonary disease	12,042 (9.8%)	30 (5.5%)	< 0.001	90 (5.8%)	30 (5.8%)	0.999
Peptic ulcer disease	12,025 (9.8%)	28 (5.1%)	< 0.001	99 (6.4%)	28 (5.4%)	0.428
Diabetes mellitus	42,643 (34.7%)	77 (14.1%)	< 0.001	209 (13.4%)	77 (14.9%)	0.419
Moderate or severe liver disease	7229 (5.9%)	24 (4.4%)	0.14	91 (5.8%)	24 (4.6%)	0.293
Chronic kidney disease	92,238 (75.0%)	130 (23.8%)	< 0.001	388 (24.9%)	130 (25.2%)	0.953
Advanced chronic kidney disease*	51,628 (42.0%)	41 (7.51%)	< 0.001	119 (7.6%)	41 (7.9%)	0.849
**In-hospital Acute Comorbidities**						
*Pulmonary complications*						
Prolonged mechanical ventilation**	10,506 (8.6%)	416 (76.2%)	< 0.001	1184 (76.0%)	387 (74.9%)	0.494
Re-intubation	5989 (4.9%)	228 (41.8%)	< 0.001	688 (44.2%)	217 (42.0%)	0.344
Acute respiratory distress syndrome	509 (0.4%)	7 (1.3%)	0.002	35 (2.3%)	7 (1.4%)	0.208
Pleural effusion	376 (0.3%)	3 (0.6%)	0.30	11 (0.7%)	3 (0.6%)	0.753
Chest tube insertion	1041 (0.9%)	111 (20.3%)	< 0.001	247 (15.9%)	90 (17.4%)	0.429
*Hemodynamic complications*						
Hypovolemic shock	668 (0.5%)	12 (2.2%)	< 0.001	32 (2.1%)	10 (1.9%)	0.856
ECMO use	147 (0.1%)	17 (3.1%)	< 0.001	44 (2.8%)	12 (2.3%)	0.532
*Main cause of infection*						
Pneumonia	2346 (1.9%)	5 (0.9%)	0.09	14 (0.9%)	5 (1.0%)	0.894
Urinary tract infection	2984 (2.4%)	4 (0.7%)	0.01	19 (1.2%)	4 (0.8%)	0.397
Severe sepsis	6041 (4.9%)	72 (13.2%)	< 0.001	243 (15.6%)	68 (13.2%)	0.166
*Outcomes*						
Index hospital stay (day) (IQR)	6 (3–11)	15 (5–34)	< 0.001	12 (5–28)	15 (5–35)	0.007
Follow-up duration (year) (IQR)				3.32 (0.1–8.85)	2.49 ()	
30-day mortality	2490 (2.0%)	99 (18.1%)	< 0.001	248 (15.9%)	95 (18.4%)	0.207
Long-term mortality	73,777 (60.0%)	134 (24.5%)	< 0.001	729 (46.8%)	129 (25.0%)	< 0.001
ESKD	32,445 (26.4%)	45 (8.2%)	< 0.001	208 (13.4%)	55 (10.6%)	0.892

ECMO, extracorporeal membrane oxygenation; ESKD, end stage kidney disease; IQR, interquartile range.

*Advanced chronic kidney disease: presence of CKD coding and concomitant reimbursement coding of erythropoiesis-stimulating agents without copayment.

**Prolonged mechanical ventilation: the use of mechanical ventilation ≥ 21 days.

To evaluate the potential etiology of AKI in our cohort (Supplemental Table 1), we collected the data of in-hospital acute comorbidities, including hypovolemic shock, cardiogenic shock, and severe sepsis which may relate to AKI. In addition, we also collected the data of contrast exposure and nephrotoxicity medications exposure before AKI-RRT after propensity score matching. Non-trauma patients had higher risk of cardiogenic shock (17.4% vs. 1.2%, *p* < 0.001) and nephrotoxicity medications exposure (18.6% vs. 2.9%, *p* < 0.001).

The overall median (IQR) follow-up duration is 2.65 (0.14–7.04) years. The vehicle-traumatic group follow-up duration is 3.32 (0.10–8.85) years and the non-traumatic group follow-up duration is 2.49 (0.16–6.39) years.

### Outcomes in patients with AKI-RRT after discharge

After propensity score matching, the follow-up period was 2.98 years. The 30-day mortality was similar between two groups (18.4% vs. 15.9%, *p* = 0.207). Although the baseline comorbidities and acute medical problems before index hospitalization were similar in both groups after matching, vehicle-traumatic AKI-RRT patients had a longer length of stay in hospital [median IQR 15 (5–35) vs. 12 (5–28) days, *p* = 0.007). The long-term incidence of ESKD was similar between groups (10.6% vs. 13.4%, *p* = 0.832) (Table 1).

The risk of developing ESKD (HR, 1.166, *p* = 0.377) and 30-day mortality after discharge (HR, 1.134, *p* = 0.301) among the vehicle-traumatic AKI-RRT patients relative to non-traumatic AKI-RRT were similar. However, vehicle-traumatic AKI-RRT patients had a lower risk of long-term mortality than non-traumatic group (adjust HR: 0.473, *p* < 0.001) (Table 2). In addition, vehicle-traumatic AKI-RRT patients was associated with lower ESKD rates (HR, 0.552; 95% CI, 0.325–0.937; *p* = 0.028) than non-traumatic AKI-RRT patients in multivariable competing risk models which set death as a competing risk. Furthermore, this lower mortality rate after vehicle-traumatic AKI-RRT persisted after adjusting for age, gender and Charlson score Cox proportional analysis (*p* < 0.001) (Fig. 3).

**Table 2:** Incidence and risks for outcome of interest in AKI patients with renal replacement therapy during hospitalization, between vehicle-traumatic patients and their matches.

Incidence							Crude		Adjust*	
	Events	Person-years	Incidence rate per 1000 person-years	Events	Person-years	Incidence rate per 1000 person-years	Hazard ratio (95% CI)	*p*	Hazard ratio (95% CI)	*p*
	Trauma			Non-trauma			Trauma vs. non-trauma			
Long-term ESKD	45	3614.99	12.45	132	11068.44	11.93	1.039 (0.741–1.457)	0.825	1.166 (0.829–1.638)	0.377
30-day mortality	95	36.94	2571.74	248	111.68	2220.63	1.154 (0.911–1.462)	0.234	1.134 (0.894–1.438)	0.301
Long-term mortality	129	2453.31	52.58	769	5946.12	129.33	0.434 (0.360–0.523)	< 0.001	0.473 (0.392–0.571)	< 0.001

ESKD, end-stage kidney disease; CI, confidence interval.

*The multivariable Cox regression model selected covariates from all variables in Table 1 and cardiogenic shock and exposure to nephrotoxic medications by a stepwise procedure.

After stratified vehicle-traumatic AKI-RRT patients by injured body part (Supplemental Table 2), the risk of developing ESKD and 30-day mortality after discharge among the vehicle-traumatic AKI-RRT patients relative to non-traumatic AKI-RRT were similar except for soft tissue injury from trauma which had lower incidence of 30-day mortality (HR, 0.714, *p* = 0.003). Bones (adjust HR: 0.580, *p* < 0.001) and abdomen injury (adjust HR: 0.607, *p* < 0.001) from trauma had lower risk of long-term mortality than non-traumatic group.

After stratified AKI-RRT patients by age (Supplemental Table 3), vehicle-traumatic AKI-RRT patients had lower risk of long-term mortality in elder (adjust HR: 0.793, *p* = 0.042) and younger (adjust HR: 0.580, *p* < 0.001) subgroup than non-trauma cohorts. However, vehicle-traumatic AKI-RRT patients had higher incidence of 30-day mortality in the elder subgroup (adjust HR: 1.441, *p* = 0.016).

## Discussion

Our study is, to our knowledge, the first to describe the short-term and long-term outcomes of vehicle-traumatic AKI-RRT patients from a large national database. Our study demonstrated that vehicle-traumatic AKI-RRT patients had lower long-term mortality than non-traumatic AKI-RRT patients. However, incident ESKD and short-term mortality were similar between vehicle-traumatic and non-traumatic AKI-RRT patients.

### The trend of vehicle-traumatic AKI-RRT

In an observational study in the United States, the incidence of AKI-RRT increased at an average of 10% per year from 2000 to 2009^9^. In the NHIRD, we found that the proportions of patients with AKI-RRT among vehicle-traumatic patients also increased throughout the years. The older the patients, the higher annual number of trauma cases was found; however, the oldest vehicle-traumatic group had the lowest incidence of AKI-RRT (Fig. 2). These results may indicate that young people had higher severity of trauma and the consequent risk of AKI-RRT. There seemed to be no difference in body distribution of injury on the incident vehicle-traumatic AKI-RRT except for the lowest incidence of soft tissue injury related AKI-RRT.

### ESKD and long-term mortality in patients with vehicle-traumatic AKI-RRT

AKI in traumatic patients is a common complication and associated with significantly higher 30-day and 1-year mortality than traumatic patients without AKI^12^. Although trauma-related risk factors for kidney injury (e.g., direct lesions to the kidneys, shock, ischemia–reperfusion, rhabdomyolysis, exposure to nephrotoxic substances, abdominal compartment syndrome, hemorrhagic shock and sepsis), The rate of long term incident ESKD in patients after AKI-RRT in our study did not significantly differ vehicle-trauma and non-trauma populations. However, compared to non-traumatic AKI-RRT patients, traumatic patients had lower risk of long-term mortality after survival in our cohort. The above results may indicate that the etiology of AKI-RRT may be associated with long-term mortality. On the other hand, the risk of ESKD existed if renal damage happened regardless of trauma or non-trauma. If we consider death as a competing risk, vehicle-traumatic AKI-RRT patients were associated with lower ESKD rates than non-traumatic AKI-RRT patients, which may indicate that vehicle-traumatic AKI-RRT patients had better renal recovery potential. In addition, the vehicle-traumatic AKI-RRT patients with soft tissue injuries may have lower severity trauma which may contribute to better 30-day mortality.

The observed decreased long-term mortality and long-term ESKD in vehicle-traumatic AKI-RRT patients after 30 days can be related to the overall better health status of these patients before the traumatic injury and that perhaps a greater burden of co-morbidity and complications in the more non-traumatic AKI-RRT population with greater baseline debility. This cannot be fully addressed with propensity score-matching involving cross-sectional comorbidity data at the time of or before index admission.

One of the few other studies examining long term-outcomes after traumatic AKI examined 40 soldiers who survived traumatic AKI-RRT in that study there was a lower incidence of ESKD (2.5% vs. 10.6%) and a lower long-term mortality (2.5% vs. 25%) rate over a median follow-up of 2.7 years than in our cohort^14^. These differences might be attributed to low incidence rates, younger age (26 ± 6 years), and fewer baseline comorbidities in the military study^14^.

### The management of vehicle-traumatic AKI

Management of AKI should be considered in the context of the underlying etiology; for instance, sepsis-associated AKI will focus on source control of infection hemodynamic support and secondary injury prevention^15^, while nephrotoxic will focus on identification removal of the offending agent^16^.

However, the management of traumatic AKI is complicated because it commonly involves multiple risk factors for AKI, including direct traumatic injury to the kidneys, hypovolemic shock, ischemia–reperfusion injury, exposure to endogenous and exogenous nephrotoxic substances, and secondary infection. Accordingly an understanding of the underlying cause of traumatic AKI is required to enable its optimal management. In addition, we have demonstrated that that traumatic AKI-RRT patients had lower long-term rates of death or ESKD in competing risk analsysis compared to non-traumatic AKI-RRT patients; this might indicate that severe traumatic AKI has a generally good prognosis justifying aggressive treatment including the use of RRT where indicated.

### Follow-up strategy in patients of AKI-RRT

AKI-RRT has been shown to be associated with an increased incidence of ESKD, mortality^17^, coronary events^13^, stroke^18^, and bone fracture^19^ among those who survived to hospital discharge. Although guidelines published by kidney disease: improving global outcomes (KDIGO) Clinical Practice Guideline recommend that survivors of AKI be followed up by a nephrologist within 90 days^20^, reports in the literature suggest only a small proportion of patients after severe AKI are followed-up by a nephrologist after survival discharge^21–23^. Given their young age, relative lack or comorbidity, and still significant risk of developing ESKD, AKI-RRT is a neglected population where focused medical follow-up could address the progression of CKD and development of other complications over a long expected lifespan. These results further provide the outcome information for patient care and rational health resource allocation.

### Limitation

Our study has certain limitations. Firstly, as all observational studies, we could not exclude the possibility of residual confounding. To decrease the effect of potential confounding factors, we developed a 3:1 propensity score. We were able to construct a comparison cohort with balanced covariates between the two groups. Second, with some administrative databases, detailed in-hospital parameters, and laboratory results were not available in our cohort. Therefore, we could not examine whether classifying patients into different severity scoring systems, such as the Injury Severity Score (ISS) and Acute Physiology and Chronic Health Evaluation score. The severity of trauma and illness has been found to be associated with AKI^8,24^, and thus could be potential predictors of clinical outcome. To attenuate the effect of the limitations, we did adjust by Charlson comorbidity index (pre-admission comorbidities) and in-hospital acute comorbidities, including pulmonary and hemodynamic complications, to evaluate the disease severity of each patient in our database. We further stratify our trauma patients based upon injured body regions as proxies to evaluate the traumatic severity. Third, we did not analyze the effect of duration and modality of RRT which may be the predictors of disease severity and the confounding factors of long-term outcomes in our study. To attenuate the effect of the limitations, we did adjust by Charlson comorbidity index (pre-admission comorbidities) and in-hospital acute comorbidities, including pulmonary and hemodynamic complications, to evaluate the disease severity of each patient in our database.

## Conclusion

In our study, we found that the vehicle-traumatic AKI-RRT patients had better long-term survival than non-traumatic counterparts with AKI-RRT. However, there were similar outcomes of ESKD and short-term mortality. Our findings are pertinent to civilian populations with similar demographics and may inform care in real-world practice.

## Methods

### Study design and setting

#### National Health Insurance registration database (NHIRD)

The Taiwan National Health Insurance (NHI) program is mandatory and universal, offering comprehensive medical care coverage to more than 99% of the country’s population of 23 million people. This nationwide compulsory healthcare program that covers outpatient visits, hospital admissions, prescriptions, interventional procedures, disease profiles, and vital statuses. Taiwan’s NHI records are regularly inspected, and physicians are subject to statutory regulation^13,19,25–28^. The National Health Insurance Research Database (NHIRD) thus represents a comprehensive, high-quality record of healthcare episodes provided to the Taiwanese population.

### Selection of participants

We enrolled patients aged ≥ 18 years who were admitted because of major trauma and developed AKI-RRT during their index admission as recognized by RRT-related procedure codes and survived to hospital discharge. All diagnoses, including trauma-related injuries and baseline comorbidities, were obtained by the codes of International Classification of Diseases, 9th Revision, Clinical Modification (ICD-9-CM). Those that were related to vehicle-related accidents, defined as any of ICD-9-CM E-Code E800 to E844 included in the diagnosis codes, were aggregated to individuals and were enrolled as study subjects^25^. When more than one accident was encountered by a single person, only the first event was counted for analysis. We identified baseline comorbidities from at least three outpatient visits or one inpatient claim within a one-year antecedent to the index admission with first dialysis. This rule was constructed based on a relatively strict criterion and was well validated with good predictive power^13,19,25,26,29^.

Dialysis patients who underwent renal transplantation, vascular access creation, or peritoneal-dialysis catheter implantation for chronic dialysis and those who had received chronic dialysis prior to the index admissions were excluded. To compare the long-term outcomes of interest, we constructed a comparator group of non-traumatic AKI-RRT patients matched to vehicle-traumatic AKI-RRT patients in a 3:1 ratio based on age, gender, and propensity scores. (Supplemental Table 4).

As all personal information is de-identified in the research database to protect privacy, no informed consent was required, and this study was deemed exempt from a full ethical review by the institutional review board of the National Taiwan University Hospital (201212021RINC).

### Outcomes

Our primary outcome was long-term all-cause mortality after hospital discharge. The secondary outcomes were 30-day all-cause mortality and de novo end-stage kidney disease (ESKD) defined as the requirement of dialysis for at least three months after hospital discharge. We used a selection period of 90 days to define ESKD because all patients receiving dialysis for more than 90 days in Taiwan can apply to the NHI for catastrophic illness registration cards^30^. Each patient was monitored from the date of discharge and was censored at either death, dialysis, or the end of the study (December 31, 2010), whichever occurred first.

We also stratified trauma patients by injured body part and age to evaluate the outcomes comparing to their matches in the subgroup analysis.

### Research variables

We recorded the disease severity and patient condition during index hospitalization. Disease severity and patient condition are estimated by the ICU procedure and complications with acute pulmonary [prolonged mechanical ventilation (the use of mechanical ventilation ≥ 21 days), re-intubation, acute respiratory distress syndrome, pleural effusion, and chest tube insertion], cardiovascular [hemopericardium, hypovolemic shock, cardiac arrest, heart block, atrial fibrillation, extracorporeal membrane oxygenation (ECMO) and intra-aortic balloon pumping (IABP)] and infectious (pneumonia, urinary tract infection and severe sepsis) and other (delirium, stroke and gastrointestinal bleeding) disorders^31^.

The Charlson comorbidity index^32^ was computed using baseline comorbidities. Additional adjustments in these models included control for direct effects from age, gender, and comorbidities that are listed in Table 1.

### Analysis

Baseline characteristics were described as the percentages for categorical variables and median with interquartile range (IQR) for continuous variables. Differences between the trauma and non-trauma related groups were compared by the independent *t-*test or χ^2^ test where appropriate.

To estimate each patient’s propensity score for vehicle-traumatic AKI-RRT, we fitted a separate multivariable logistic regression model with the factors predicting vehicle trauma as admitting diagnosis in patients with AKI-RRT during index hospitalization^33^ (further seen in Supplemental Table 4). The caliper distance is 0.25 and subjects are matched without replacement in this propensity score matching. The estimated propensity score was also added to adjusting the Cox regression model as a single covariate for controlling selection bias. Multivariable logistic regression models before and after propensity-scored matching were applied to estimate odds ratio (OR) of study outcomes after adjusting all the confounders predicting trauma (Supplemental Table 4).

We evaluated the association of vehicle-traumatic AKI-RRT with the risks of long-term ESKD, 30-day mortality and long-term mortality among our cohort using Cox regression models with time-dependent covariates. To assess multivariate Cox regression models in mortality and ESKD analysis, the relationship between scaled Schoenfeld residuals and time was examined, with no significant evidence for non-proportionality (*p* value > 0.05), therefore we accepted the assumption in the Cox model of constant hazard ratios over time.

The significance levels for entry and stay were set to 0.15 to be conservative. Then, with the aid of substantive knowledge, the best candidate final logistic model was identified manually by dropping the covariates with *p* value > 0.05 one at a time until all regression coefficients were significantly different from 0.

All the analyses were conducted with R software, version 2.8.1 (Free Software Foundation, Inc., Boston, MA, USA); competing-risk analysis was performed using Stata/MP version 12 (Stata Corporation). A two-sided p-value < 0.05 was considered to be statistically significant.

## Supplementary information

Supplementary Information.
