# Supplementary material for: Long-term outcomes following vehicle trauma related acute kidney injury requiring renal replacement therapy: a nationwide population study
Source: Sci Rep. 2020 Nov 25;10:20572. doi: 10.1038/s41598-020-77556-3 (PMC7689526; doi:10.1038/s41598-020-77556-3)
Supplement: Supplementary file 1 — Supplementary Information. [file 41598_2020_77556_MOESM1_ESM.docx]

|  | Non-trauma group  (n = 1,551) | Trauma group  (n = 517) | *p*-Value |
| --- | --- | --- | --- |
| Severe sepsis | 243 (15.6%) | 68 (13.2%) | 0.166 |
| Cardiogenic shock | 271(17.40%) | 6 (1.2%) | <0.001 |
| Hypovolemic shock | 32 (2.1%) | 10 (1.9%) | 0.856 |
| Contrast exposure | 237(15.28%) | 63(12.18%) | 0.084 |
| Nephrotoxicity medications | 288(18.56%) | 15(2.90%) | <0.001 |

Supplemental Table 1 AKI associated risk factors in the vehicle-traumatic AKI-RRT group and non-traumatic AKI-RRT group.

Supplemental Table 2. Incidence and risks for outcome of interest in AKI patients with renal replacement therapy during hospitalization, between vehicle-traumatic patients and their matched counterpart stratified by injured body part.

| Incidence |  |  |  | | |  |  |  | | Crude |  | Adjust* |  |
| --- | --- | --- | --- | --- | --- | --- | --- | --- | --- | --- | --- | --- | --- |
|  | Events | Person-Years | Incidence Rate per 1000 Person-Years |  | Events | Person-Years | Incidence Rate per 1000 Person-Years |  |  | Hazard Ratio  (95% CI) | p | Hazard Ratio  (95% CI) | p |
|  | Trauma (injured body part: brain) | | |  | Non-trauma | | |  |  | Trauma vs Non-trauma | | | |
| Long–term ESKD | 23 | 7,192 | 3.20 |  | 132 | 11068.44 | 11.93 |  | 1.278  (0.821-1.990) | | 0.276 | 1.300  (0.835-2.024) | 0.245 |
| 30-day mortality | 44 | 1,372 | 32.07 |  | 248 | 111.68 | 2220.63 |  | 1.275  (0.926-1.756) | | 0.136 | 1.270  (0.922-1.749) | 0.143 |
| Long-term mortality | 66 | 48,498 | 1.36 |  | 769 | 5946.12 | 129.33 |  | 0.744  (0.578-0.959) | | 0.022 | 0.791 (0.614-1.019) | 0.069 |
|  | Trauma (injured body part: bones) | | |  | Non-trauma | | |  | | Trauma vs Non-trauma | | | |
| Long–term ESKD | 25 | 8,967 | 2.79 |  | 132 | 11068.44 | 11.93 |  | 0.891  (0.581-1.365) | | 0.596 | 0.908 (0.593-1.393) | 0.660 |
| 30-day mortality | 49 | 1,277 | 38.37 |  | 248 | 111.68 | 2220.63 |  | 0.905  (0.667-1.229) | | 0.523 | 0.925 (0.681-1.256) | 0.619 |
| Long-term mortality | 83 | 59,784 | 1.39 |  | 769 | 5946.12 | 129.33 |  | 0.518  (0.412-0.652) | | <0.001 | 0.580 (0.461-0.731) | <0.001 |
|  | Trauma (injured body part: chest) | | |  | Non-trauma | | |  | | Trauma vs Non-trauma | | | |
| Long–term ESKD | 8 | 69 | 115.28 |  | 132 | 11068.44 | 11.93 |  | 0.743  (0.366-1.510) | | 0.412 | 0.770  (0.379-1.567) | 0.471 |
| 30-day mortality | 21 | 567 | 37.04 |  | 248 | 111.68 | 2220.63 |  | 0.993  (0.639-1.544) | | 0.975 | 0.966  (0.621-1.503) | 0.966 |
| Long-term mortality | 34 | 22,331 | 1.52 |  | 769 | 5946.12 | 129.33 |  | 0.679  (0.479-0.963) | | 0.030 | 0.726  (0.512-1.029) | 0.072 |
|  | Trauma (injured body part: abdomen) | | |  | Non-trauma | | |  | | Trauma vs Non-trauma | | | |
| Long–term ESKD | 23 | 5,508 | 4.18 |  | 132 | 11068.44 | 11.93 |  | 0.890  (0.575-1.379) | | 0.603 | 0.969 (0.625-1.503) | 0.888 |
| 30-day mortality | 48 | 1,373 | 34.96 |  | 248 | 111.68 | 2220.63 |  | 0.967  (0.713-1.312) | | 0.830 | 0.986 (0.726-1.338) | 0.927 |
| Long-term mortality | 71 | 52,757 | 1.35 |  | 769 | 5946.12 | 129.33 |  | 0.524  (0.410-0.671) | | <0.001 | 0.607 (0.474-0.777) | <0.001 |
|  | Trauma (injured body part: soft tissue) | | |  | Non-trauma | | |  | | Trauma vs Non-trauma | | | |
| Long–term ESKD | 10 | 2,312 | 4.33 |  | 132 | 11068.44 | 11.93 |  | 0.854  (0.696-1.048) | | 0.130 | 0.845  (0.688-1.037) | 0.107 |
| 30-day mortality | 27 | 685 | 39.42 |  | 248 | 111.68 | 2220.63 |  | 0.668  (0.535-0.835) | | <0.001 | 0.714  (0.571-0.894) | 0.003 |
| Long-term mortality | 25 | 25,684 | 0.97 |  | 769 | 5946.12 | 129.33 |  | 0.934  (0.748-1.167) | | 0.549 | 0.910  (0.997-1.004) | 0.841 |

**Abbreviations:** ESKD, end-stage kidney disease; CI, confidence interval.

* The multivariable Cox regression model selected covariates from all variables in Table1 by a stepwise procedure.

Supplemental Table 3. Incidence and risks for outcome of interest in AKI patients with renal replacement therapy during hospitalization, between vehicle-traumatic patients and their counterpart stratified by age.

| Incidence |  |  |  | | |  |  |  | | Crude |  | Adjust* |  |
| --- | --- | --- | --- | --- | --- | --- | --- | --- | --- | --- | --- | --- | --- |
|  | Events | Person-Years | Incidence Rate per 1000 Person-Years |  | Events | Person-Years | Incidence Rate per 1000 Person-Years |  |  | Hazard Ratio  (95% CI) | p | Hazard Ratio  (95% CI) | p |
|  | Trauma (age≥50-year-old) | | |  | Non-trauma | | |  |  | Trauma vs Non-trauma | | | |
| Long–term ESKD | 32 | 6,151 | 5.20 |  | 132 | 11068.44 | 11.93 |  | 1.707  (1.164-2.503) | | 0.006 | 1.267  (0.846-1.898) | 0.250 |
| 30-day mortality | 61 | 1,580 | 38.61 |  | 248 | 111.68 | 2220.63 |  | 1.598  (1.211-2.106) | | 0.001 | 1.441  (1.071-1.939) | 0.016 |
| Long-term mortality | 94 | 55,160 | 1.70 |  | 769 | 5946.12 | 129.33 |  | 1.286  (1.035-1.597) | | 0.023 | 0.793  (0.635-0.992) | 0.042 |
|  | Trauma (age<50-year-old) | | |  | Non-trauma | | |  | | Trauma vs Non-trauma | | | |
| Long–term ESKD | 13 | 5,753 | 2.26 |  | 132 | 11068.44 | 11.93 |  | 0.891  (0.581-1.365) | | 0.596 | 0.908  (0.593-1.393) | 0.660 |
| 30-day mortality | 31 | 939 | 33.01 |  | 248 | 111.68 | 2220.63 |  | 0.905  (0.667-1.229) | | 0.523 | 0.925  (0.681-1.256) | 0.619 |
| Long-term mortality | 34 | 23,369 | 1.45 |  | 769 | 5946.12 | 129.33 |  | 0.518  (0.412-0.652) | | <0.001 | 0.580  (0.461-0.731) | <0.001 |

**Abbreviations:** ESKD, end-stage kidney disease; CI, confidence interval.

* The multivariable Cox regression model selected covariates from all variables in Table1 by a stepwise procedure.

Supplemental Table 4. The risk factors for vehicle-traumatic AKI-RRT in order to minimize residual confounding effects in the matching process to non-traumatic AKI-RRT.

| Items | OR | lower 95% CI | upper  95% CI | *p* |
| --- | --- | --- | --- | --- |
| Age (year) | 1.035 | 1.03 | 1.041 | <0.001 |
| Gender | 1.282 | 1.026 | 1.602 | 0.029 |
| Cerebrovascular disease | 2.047 | 1.1 | 3.811 | 0.024 |
| Rheumatologic disease | 3.029 | 1.322 | 6.937 | 0.009 |
| Advanced CKD | 0.617 | 0.432 | 0.883 | 0.008 |
| PMV | 0.473 | 0.378 | 0.592 | <0.001 |
| ECMO | 0.386 | 0.189 | 0.79 | 0.009 |

Hosmer-Lemeshow goodness of fit [GOF] test p=0.228, AUC= 0.698

This logistic regression model was adjusted for age, gender, congestive heart failure, CVA, CKD, COPD, coronary artery disease, dementia, diabetes Mellitus, hemiplegia, liver disease, peptic ulcer, peripheral vascular disease, solid tumor, SLE, atrial fibrillation, dyslipidemia

**Abbreviations;** AKI-RRT, acute kidney injury with renal replacement therapy; CI, confidence interval; CKD, chronic kidney disease, COPD, chronic obstructive pulmonary; CVA, cerebral vascular accident; ECMO, extracorporeal membrane oxygenation; PMV, prolonged mechanical ventilation
